# Clinical Insights Into Novel Immune Checkpoint Inhibitors

**DOI:** 10.3389/fphar.2021.681320

**Published:** 2021-05-06

**Authors:** Jii Bum Lee, Sang-Jun Ha, Hye Ryun Kim

**Affiliations:** ^1^ Division of Hemato-oncology, Wonju Severance Christian Hospital, Yonsei University Wonju College of Medicine, Wonju, South Korea; ^2^ Division of Medical Oncology, Department of Internal Medicine, Yonsei Cancer Center, Yonsei University College of Medicine, Seoul, South Korea; ^3^ Department of Biochemistry, College of Life Science & Biotechnology, Yonsei University, Seoul, South Korea

**Keywords:** immune checkpoint, LAG-3, TIGIT, TIM-3, B7-H3, VISTA, ICOS, BTLA

## Abstract

The success of immune checkpoint inhibitors (ICIs), notably anti-cytotoxic T lymphocyte associated antigen-4 (CTLA-4) as well as inhibitors of CTLA-4, programmed death 1 (PD-1), and programmed death ligand-1 (PD-L1), has revolutionized treatment options for solid tumors. However, the lack of response to treatment, in terms of *de novo* or acquired resistance, and immune related adverse events (IRAE) remain as hurdles. One mechanisms to overcome the limitations of ICIs is to target other immune checkpoints associated with tumor microenvironment. Immune checkpoints such as lymphocyte activation gene-3 (LAG-3), T cell immunoglobulin and ITIM domain (TIGIT), T cell immunoglobulin and mucin-domain containing-3 (TIM-3), V-domain immunoglobulin suppressor of T cell activation (VISTA), B7 homolog 3 protein (B7-H3), inducible T cell costimulatory (ICOS), and B and T lymphocyte attenuator (BTLA) are feasible and promising options for treating solid tumors, and clinical trials are currently under active investigation. This review aims to summarize the clinical aspects of the immune checkpoints and introduce novel agents targeting these checkpoints.

## Background

Cancer cells have characteristics that allow diversification and sustenance of their neoplastic state (Hanahan and Weinberg, 2011). One of the hallmarks of cancer is immune evasion; cancer cells hamper immune activation by limiting T cell activation and expressing immune checkpoint proteins on T cells (Vinay et al., 2015). Blocking cytotoxic T lymphocyte associated antigen-4 (CTLA-4) and the interaction between programmed death 1 (PD-1) and programmed death ligand-1 (PD-L1) elicit activation of the host immune system through T cell responses (Pardoll, 2012). These findings have led to the development of immune checkpoint inhibitors (ICIs) to control one of the key mechanisms utilized by cancer cells (Pardoll, 2012). In 2011, ipilimumab, the first anti-CTLA-4 monoclonal antibody (mAb), was approved for treating metastatic melanoma (Cameron et al., 2011). Thereafter, anti-PD-1 mAbs such as pembrolizumab, nivolumab, cemiplimab and as well as anti-PD-L1 mAbs such as atezolizumab, avelumab, durvalumab, have been used to treat patients with cancer, especially in locally advanced and metastatic settings (Qin et al., 2019; Vaddepally et al., 2020). Besides PD-L1 expression, several emerging biomarkers have gained wide attention (Darvin et al., 2018). Pembrolizumab was approved in solid tumors harboring microsatellite instability-high (MSI-H) or mismatch repair deficient (dMMR), and high tumor mutation burden (TMB-H) defined as 10 mutations/megabase based on FoundationOneCDx assay (Foundation Medicine, Inc.) (Marcus et al., 2019; Marabelle et al., 2020).

Despite the feasibility and anti-tumor activity of ICIs, there remain several hurdles in immunotherapy for cancer. Only a subset of patients respond to treatment, and the majority of patients who have durable responses eventually experience disease progression (Trebeschi et al., 2019). Furthermore, patients experience IRAE, some of which are highly toxic (Boutros et al., 2016; Wang et al., 2018). To overcome these impediments, treatment strategies such as combination with chemotherapy, targeted agents, or radiotherapy have been implemented (Gandhi et al., 2018; Wang et al., 2018; Rini et al., 2019). Notably, treatment with a combination of different ICIs has resulted in increased clinical responses, as observed with the combination of nivolumab and ipilimumab in melanoma, non-small cell lung cancer (NSCLC), and renal cell carcinoma (RCC) (Rizvi et al., 2016; Hellmann et al., 2018; Motzer et al., 2018).

Promising results from the combination of anti-CTLA-4 and PD-L1 mAbs have resulted in the launch of several other ICI combinations with non-overlapping mechanisms of action that may increase efficacy and minimize toxicity (Barbari et al., 2020). Currently, approximately 2/3 of all oncology trials are dedicated to T cell-targeting immunomodulators, and there are more than 3,000 ongoing clinical trials (Xin Yu et al., 2019).

Resistance to immunotherapy is associated with loss of immunogenic neoantigens, increase of immunosuppressive cells, and upregulation of alternate immune checkpoint receptors (Sharma et al., 2017). This review provides an overview of the mechanisms and ongoing clinical trials specifically on novel emerging immune checkpoints, including lymphocyte activation gene-3 (LAG-3), T cell immunoglobulin and ITIM domain (TIGIT), T cell immunoglobulin and mucin-domain containing-3 (TIM-3), V-domain immunoglobulin suppressor of T cell activation (VISTA), B7 homolog 3 protein (B7-H3), inducible T cell costimulatory (ICOS), and B and T lymphocyte attenuator (BTLA) (Chapoval et al., 2001; Monney et al., 2002; Yu et al., 2009; Paulos and June, 2010; Wang et al., 2011; Andrews et al., 2017; Marinelli et al., 2018).

## LAG-3

LAG-3 is a protein comprising four partsthe hydrophobic, extracellular, transmembrane, and cytoplasmic domains. LAG-3 shares structural similarity with CD4 in having four extracellular regions (Triebel et al., 1990; Huard et al., 1997). It is expressed mainly on activated CD4^+^ and CD8^+^ T cells, regulatory T cells (Tregs), and natural killer (NK) cells, as well as on B cells and plasmacytoid dendritic cells (DCs) (Table 1) (Huard et al., 1995; Andreae et al., 2002; Huang et al., 2004; Kisielow et al., 2005). LAG-3 binds its canonical ligand, major histocompatibility complex class II (MHC-II), as well as other ligands, including galectin-3, LSECtin, -synuclein, and fibrinogen-like protein 1 (FGL1), thereby inducing exhaustion of immune cells and decreased cytokine secretion (Baixeras et al., 1992; Huard et al., 1994; Kouo et al., 2015; Anderson et al., 2016; Baumeister et al., 2016; Mao et al., 2016; Wang et al., 2019).

**TABLE 1:** Overview of novel immune checkpoints.

**Immune checkpoints**	**LAG-3**	**TIGIT**	**TIM-3**	**B7-H3**	**VISTA**	**ICOS**	**BTLA**
**Other names**	CD223	Vstm3, Vsig9, WUCAM	HAVCR2	CD276	Dies1, DD1, Gi24, B7-H5, PD-1H	CD278	CD272
**Function**	Co-inhibition	Co-inhibition	Co-inhibition	Co-inhibition or co-stimulation	Co-inhibition	Co-inhibition or co-stimulation	Co-inhibition or co-stimulation
**Cells that express the immune checkpoints**	NK cells, DC, activated T cells, Tregs, B cells,	NK cells, T cells	NK cells, DCs, activated T cells, Tregs, B cells, monocytes, cancer cells	NK cells, DCs, activated T cells, monocytes, cancer cells	T cells, myeloid cells	Activated T cells	Mature T cells, Tregs, B cells, macrophages
**Ligands or receptors**	MHC-II, galectin-3, LSECtin, a-synuclein, FGL1	CD155, CD112	HMGB-1, galectin-9, ceacam-1, PtdSer	Unknown	VSIG-3	ICOSL	HVEM, LIGHT, lymphotoxin-
**Immune checkpoint agents**	APC activator, anti-LAG3 mAb, LAG3 and PD1 DART protein, LAG3 fusion protein, bispecific Ab to both LAG3 and PD-L1	Anti-TIGIT mAb	Anti-TIM-3 mAb, anti-PD-1/TIM3 bispecific Ab	Anti-B7-H3 mAb, B7-H3-targeting ADC, radiolabeled anti-B7-H3 mAb, CAR T-cell therapy	Anti-VISTA mAb, small molecule VISTA	Anti-ICOS agonist, anti-ICOS antagonist	
**No. of investigational agents**	17	10	8	11	3	4	4
**Clinical trials**							
Phase 1	Completed (eftilagimod alpha, BI 754111, Sym022, INCAGN02385), ongoing	Ongoing	Completed (Sym023), ongoing	Completed (enoblituzumab), ongoing	Completed (CA-170), ongoing	Ongoing	Completed (JTX-2011), ongoing
Phase 2	Completed (eftilagimod alpha, LAG525), ongoing	Ongoing	Ongoing	Ongoing	NA	NA	NA
Phase 3	Ongoing (MGD013)	Ongoing (tiragolumab)	Ongoing (sabatolimab)	Ongoing	NA	NA	NA
**Combination treatment**	Yes	Yes	Yes	Yes	No	Yes	Yes
Other immune checkpoint inhibitors	Yes	Yes	Yes	Yes		Yes	Yes
Targeted agents	Yes	Yes	Yes	Yes		Yes	Yes
Chemotherapy	Yes	Yes	Yes	Yes		Yes	No
Radiotherapy	Yes	No	No	Yes		No	No

**Abbreviations:** APC, antigen presenting cell; BTLA, B and T-lymphocyte attenuator; CAR-T, chimeric antigen receptor T cell; DART, dual-affinity re-targeting proteins; DCs, dendritic cells; Dies 1, differentiation of embryonic stem cells 1; HAVCR2, hepatitis A virus cellular receptor 2; HVEM, herpes-virus entry mediator; mAb, monoclonal antibody; ICOS, Inducible T cell costimulator; ICOSL, Inducible T cell costimulatory ligand; LAG-3, lymphocyte-associated gene 3; NK cells, natural killer cells; PD-1H, PD-1 homologue; PD-L1, programmed death-ligand 1; PtdSer, phosphatidyl serine; T regs, ceacam-1, carcinoembryonic antigen cell adhesion molecule 1; T regs, regulatory T cells; TIGIT, T cell immunoglobulin and ITIM domain; TIM-3, T-cell immunoglobulin and mucin domain-3; VISTA, V-domain immunoglobulin suppressor of T cell activation; VSIG-3, V-Set and Immunoglobulin domain containing 3; WUCAM, Washington University cell adhesion molecule.

LAG-3 was found to be simultaneously co-expressed with other targets, such as PD-L1, TIGIT, and TIM-3, in preclinical settings (Woo et al., 2012; Baumeister et al., 2016). Blocking LAG-3 alone did not restore T cell exhaustion; however, the combination of LAG-3/PD-1 blockade resulted in reduced tumor volume (Woo et al., 2012). These findings were consistent across *in vivo* studies using murine models of other tumors, including melanoma, ovarian cancer, and lymphoma (Goding et al., 2013; Huang et al., 2015).

In humans, LAG-3 is expressed on CD8^+^ tumor-infiltrating lymphocytes (TILs) and peripheral Tregs (Camisaschi et al., 2010; Matsuzaki et al., 2010; Li et al., 2013; Llosa et al., 2015; Taube et al., 2015). CD8^+^ TILs isolated from tumors such as hepatocellular carcinoma (HCC), melanoma, ovarian cancer, and microsatellite instability high (MSI) colorectal cancer (CRC), have high levels of both PD-1 and LAG-3 (Matsuzaki et al., 2010; Li et al., 2013; Llosa et al., 2015; Taube et al., 2015). Peripheral Tregs have been observed in melanoma and CRC (Camisaschi et al., 2010). In patients with hormone receptor-positive breast cancer, treated with immunotherapy, soluble LAG-3 (sLAG-3) detected in the serum was correlated with better prognosis in terms of disease-free survival (DFS) and overall survival (OS) (Triebel et al., 2006). However, the mechanism of sLAG-3 has yet to be identified (Li et al., 2007).

### Clinical Trials on LAG-3

Co-expression of LAG-3 with immune checkpoints, such as PD-1, and robust clinical data on the efficacy of LAG-3 and PD-1 dual blockade have prompted trials focusing on this combination as well as other immune checkpoint inhibitors. Currently, there are 17 agents targeting LAG-3 (Table 2)**,** with multiple combinations of treatments across various tumors (Table 3). Eight of these agents have interim or final clinical results, and nine of the investigational agents are ongoing clinical trials.

**TABLE 2:** Emerging immune checkpoint inhibitors and their mechanisms.

**Target**	**Name of agent**	**Company**	**Mechanism**
**LAG-3**			
	Eftilagimod alpha (IMP321)	Immutep	APC activator
	Relatlimab (BMS-986016)	Bristol-Myers Squibb	IgG4 mAb
	LAG525	Norvatis	IgG4 mAb
	Cemiplimab (REGN3767)	Regeneron	mAb
	BI 754111	Bohringer Ingelheim	mAb
	Sym022	Symphogen	Fc-inert mAb
	MGD013	MacroGenics	LAG-3 and PD1 DART protein
	Mavezelimab (MK-4280)	Merck	IgG4 mAb
	TSR-033	Tesaro	IgG4 mAb
	INCAGN02385	Incyte	Fc engineered IgG1k antibody
	EOC202	EddingPharm Oncology	LAG-3 fusion protein
	89Zr-DFO-REGN3767	Memorial Sloan Kettering Cancer Center	Anti-LAG-3 antibody labeled with 89Zr
	XmAb22,841	Xencor	Bispecific antibody to both LAG3 and CTLA-4
	LBL-007	Nanjing Leads Biolabs Co	AlphaLAG-3 mAb
	FS118	F-star	Bispecific antibody to both LAG3 and PD-L1
	RO7247669	Hoffmann-La Roche	Bispecific antibody to both LAG3 and PD-L1
	EMB-02	Shanghai EpimAb Biotherapeutics	Bispecific antibody to both LAG3 and PD-L1
**TIGIT**			
	Tiragolumab (MTIG7192A/RG-6058)	Genentech	Anti-TIGIT mAb
	Vibostolimab (MK-7684)	Merck	Anti-TIGIT mAb
	Etigilimab (OMP-313M32)	OncoMed	Anti-TIGIT mAb
	BMS-986207	Bristol-Myers Squibb	Anti-TIGIT mAb
	Domvanalimab (AB-154)	Arcus Biosciences	Anti-TIGIT mAb
	ASP-8374	Potenza	Anti-TIGIT mAb
	IBI939	Innovent Biologics	Anti-TIGIT mAb
	BGB-A1217	BeiGene	Anti-TIGIT mAb
	COM902	Compugen	Anti-TIGIT mAb
	M6223	EMD Serono	Anti-TIGIT mAb
**TIM-3**			
	Sym023	Symphogen	Anti-TIM-3 mAb
	LY3321367	Eli Lilly and Company	Anti-TIM-3 mAb
	Cobolimab (TSR-022)	Tesaro	Anti-TIM-3 mAb
	Sabatolimab (MBG453)	Novartis	Anti-TIM-3 mAb
	INCAGN2390	Incyte	Anti-TIM-3 mAb
	BMS-986258	Bristol-Myers Squibb	Anti-TIM-3 mAb
	SHR-1702	Jiangsu HengRui	Anti-TIM-3 mAb
	RO7121661	Roche	Anti-PD-1/TIM-33 bispecific Ab
**B7-H3**			
	Enoblituzumab (MGA271)	MacroGenetics	Anti-B7-H3 mAb
	DS-7300a	Daiichi Sankyo	B7-H3-targeting ADC
	Orlotamab (MGD009)	MacroGenetics	B7-H3 and CD3 DART protein
	131I-Omburtamab	Y-mAbs Therapeutics	Radiolabeled anti-B7-H3 mAb
	124I-Omburtamab	Y-mAbs Therapeutics	Radiolabeled anti-B7-H3 mAb
	177Lu-DTPA-Omburtamab	Y-mAbs Therapeutics	Radiolabeled anti-B7-H3 mAb
	4SCAR-276	Shenzhen Geno-Immune Medical Institute	CAR T-cell therapy
	SCRI-CARB7H3	Seattle Childrens Hospital	CAR T-cell therapy
	B7-H3 CAR-T	BoYuan RunSheng Pharma	CAR T-cell therapy
	CAR.B7-H3	UNC Lineberger Comprehensive Cancer Center	CAR T-cell therapy
	Second-generation 4-1BB B7H3-EGFRt-DHFR	Seattle Childrens Hospital	CAR T-cell therapy
**VISTA**			
	JNJ-61610588	Johnson & Johnson	Anti-VISTA mAb
	CI-8993	Curis	Anti-VISTA mAb
	CA-170	Curis	Small molecule targeting VISTA and PD-L1
**ICOS**			
	GSK3359609	GlaxoSmithKline	Anti-ICOS agonist
	JTX-2011	Jounce Therapeutics	Anti-ICOS agonist
	MEDI-570	National Cancer Institute	Anti-ICOS antagonist
	KY1044	Kymab Limited	Anti-ICOS antagonist
**BTLA**			
	INBRX-106	Inhibrx	Hexavalent OX40 agonist Ab
	PF-04518600	Pfizer	OX40 agonist
	Cudarolimab (IBI101)	Innovent Biologics	Anti-OX40 mAb
	TAB004 (JS004)	Shanghai Junshi Bioscience	Anti-BTLA mAb

**Abbreviations:** ADC, antibody drug conjugate; APC, antigen-presenting cell; BTLA, B and T-lymphocyte attenuator; CAR, chimeric antigen receptor; CTLA-4, cytotoxic T-lymphocyte-associated protein; DART, dual-affinity re-targeting proteins; ICOS, inducible T-cell costimulator; LAG3, lymphocyte-associated gene 3; mAb, monoclonal antibody; PD-L1, programmed death-ligand 1; TIGIT, T cell immunoglobulin and ITIM domain; TIM, T-cell immunoglobulin and mucin domain-3; VISTA, V-domain immunoglobulin suppressor of T cell activation.

**TABLE 3:** Clinical trials on novel immune checkpoint inhibitors.

**Target**	**Drug**	**Clinical trial no.**	**Phase**	**Settings**	**Tumor types**	**Treatment arms**	**Status**
**LAG-3**	Eftilagimod alpha (IMP321)	NCT03252938	1	Advanced/metastatic	Solid tumors	Eftilagimod alpha	Active, not recruiting
		NCT00351949	1	Advanced/metastatic	RCC	Eftilagimod alpha	Completed
		NCT00349934	1	First line	Breast cancer	Eftilagimod alpha	Completed
		NCT02614833	2	Advanced/metastatic	Breast cancer	Eftilagimod alpha	Active, not recruiting
		NCT00324623	1	Advanced/metastatic	Melanoma	Cyclophosphamide, fludarabine followed by melan-A VLP vaccine and eftilagimod alpha	Completed
		NCT00365937	1,2	Adjuvant	Melanoma	Eftilagimod alphaHLA-A2 peptides	Terminated
		NCT01308294	1,2	Stage II-IV	Melanoma	Eftilagimod alpha+tumor antigenic peptides+monatide	Terminated
		NCT00732082	1	Advanced/metastatic	Pancreatic cancer	Eftilagimod alpha+gemcitabine	Terminated
		NCT02676869	1	Stage III-IV	Melanoma	Eftilagimod alpha+pembrolizumab	Completed
		NCT03625323	2	Advanced/metastatic	NSCLC and HNSCC	Eftilagimod alpha+pembrolizumab	Recruiting
	Relatlimab (BMS-986016)	NCT02966548	1	Advanced/metastatic	Solid tumors	Relatlimabnivolumab	Recruiting
		NCT01968109	1,2	First, second line	Solid tumors	Relatlimabnivolumab	Recruiting
		NCT03623854	2	Advanced/metastatic	Chordoma	Relatlimab+nivolumab	Recruiting
		NCT03743766	2	Advanced/metastatic	Melanoma	Relatlimab+nivolumab	Recruiting
		NCT03470922	2,3	Advanced/metastatic	Melanoma	Relatlimabnivolumab	Recruiting
		NCT03642067	2	Advanced/metastatic	MSS CRC	Relatlimab+nivolumab	Recruiting
		NCT04658147	1	Resectable	HCC	Relatlimabnivolumab	Not yet recruiting
		NCT02061761	1,2	Advanced/metastatic	Hematologic malignancies	Relatlimab+nivolumab	Active, not recruiting
		NCT04567615	2	Advanced/metastatic	HCC	Relatlimab+nivolumab	Not yet recruiting
		NCT03607890	2	Advanced, prior PD-(L)1 inhibitor	MSI-H solid tumors	Relatlimab+nivolumab	Recruiting
		NCT04326257	2	Advanced, prior PD-(L)1 inhibitor	HNSCC	Relatlimab+nivolumab or ipilimumab	Recruiting
		NCT03493932	1	Recurrent	Glioblastoma	Relatlimab+nivolumab	Recruiting
		NCT02658981	1	Recurrent	Glioblastoma	Relatlimabnivolumab or urelumab (anti-CD137)	Active, not recruiting
		NCT03610711	1,2	Advanced/metastatic	GC, GEJ cancer	Relatlimabnivolumab	Recruiting
		NCT03044613	1	Stage II/III	GC, GEJ cancer	Nivolumab, carboplatin, paclitaxel, radiationrelatlimab	Recruiting
		NCT03662659	2	Advanced/metastatic	GC, GEJ cancer	Relatlimab or nivolumabinvestigator's choice of chemotherapy	Active, not recruiting
		NCT03335540	1,2	Advanced/metastatic	Solid tumors	Relatlimab+nivolumab or cabiralizumab or ipilimumab or IDO1 inhibitor or radiation therapy	Recruiting
		NCT04611126	1,2	Advanced/metastatic	Ovarian cancer	Relatlimab, nivolumab, cyclophosphamide, fludarabine phosphate, tumor infiltrating lymphocytes infusion ipilimumab	Not yet recruiting
		NCT02488759	1,2	Neoadjuvant and metastatic	Virus-associated tumors	Nivolumabrelatlimab or ipilimumab or daratumumab	Active, not recruiting
		NCT02519322	2	Neoadjuvant and adjuvant	Melanoma	Nivolumabrelatlimab or ipilimumab	Recruiting
		NCT03459222	2	Advanced/metastatic	Solid tumors	Relatlimab, nivolumabipilimumab	Recruiting
		NCT02996110	2	Advanced/metastatic	RCC	Nivolumab+ipilimumab or BMS-986205 (IDO1i) or BMS-813160 (CCR2/5 dual antagonist)	Recruiting
		NCT02935634	2	Advanced/metastatic	GC, GEJ cancer	Nivolumabrelatlimab or ipilimumab or rucaparib or BMS-986205; ipilimumab+ucaparib; nivolumab+ipilimumab+rucaparib	Recruiting
		NCT02750514	2	Advanced/metastatic	NSCLC	Nivolumab relatlimab or ipilimumab or BMS-986205 or dasatinib	Active, not recruiting
		NCT02060188	2	Advanced/metastatic	CRC	Nivolumabrelatimab or daratumumab or ipilimumabcobimetinib	Active, not recruiting
		NCT04150965	1,2	Advanced/metastatic	Multiple myeloma	Relatlimabpomalidromide and dexamethasone; BMS-986207 (anti-TIGIT)pomalidromide and dexamethasone; elotuzumab	Recruiting
	LAG525	NCT02460224	1,2	Advanced/metastatic	Solid tumors	LAG525spartalizumab (PDR001)	Active, not recruiting
		NCT03365791	2	Advanced/metastatic	Solid or hematologic malignancy	LAG525+spartalizumab	Completed
		NCT03742349	1	Advanced/metastatic	TNBC	LAG525+spartalizumab+NIR178 or capmatinib or lacnotuzumab (MCS110) or canakinumab	Recruiting
		NCT03499899	2	Advanced/metastatic	TNBC	LAG525spartalizumabcarboplatin; LAG525+carboplatin	Active, not recruiting
		NCT03484923	2	Advanced/metastatic	Melanoma	Spartalizumab+lag525 or ribociclib or canakinumab or capmatinib	Recruiting
	Cemiplimab (REGN3767)	NCT03005782	1	Advanced/metastatic	Solid tumors or lymphomas	REGN3767cemiplimab (REGN2810)	Recruiting
	BI 754111	NCT03433898	1	Advanced/metastatic	Solid tumors	BI 754111BI 754091 (anti-PD-1)	Recruiting
		NCT03156114	1	Advanced/metastatic	Solid tumors	BI 754111+BI 754091	Active, not recruiting
		NCT03780725	1	Advanced/metastatic	NSCLC and HNSCC	BI 754111+BI 754091	Completed
		NCT03697304	2	Advanced/metastatic	Solid tumors	BI 754111 or BI 836880 (bispecific VEGF and Ang2 Ab)+BI 754091 (anti-PD-1)	Recruiting
		NCT03964233	1	Advanced/metastatic	Solid tumors	BI 754111+BI 754091BI 907828 (MDM2-p53 antagonist)	Recruiting
	Sym022	NCT03489369	1	Advanced/metastatic	Solid tumors or lymphomas		Completed
	MGD013	NCT03219268	1	Advanced/metastatic	Solid or hematologic malignancy	MGD013+margetuximab (anti-HER2 monoclonal antibody)	Recruiting
		NCT04082364	2,3	Advanced/metastatic	GC, GEJ cancer	margetuximab+INCMGA00012 (anti-PD-1); margetuximab+chemotherapyMGD013 or INCMGA00012; trastuzumab+chemotherapy (XELOX or mFOLFOX-6)	Recruiting
	Mavezelimab (MK-4280)	NCT03598608	1,2	Measurable disease	Hematologic malignancies	MK-4280+pembrolizumab	Recruiting
		NCT02720068	1	Advanced/metastatic	Solid tumors	MK4280+pembrolizumabFOLFIRI or mFOLFOX7 or lenvatinib	Recruiting
		NCT03516981	2	First line	NSCLC	MK4280+pembrolizumab or lenvatinib or quavonlimab (MK-1308)	Recruiting
	TSR-033	NCT03250832	1	Advanced/metastatic	Solid tumors	TSR-033dostarlimab (TSR-042)mFOLFOX or FOLFIRI	Recruiting
	IN-CAGN02385	NCT03538028	1	Advanced/metastatic	Solid tumors		Completed
		NCT04370704	1,2	Advanced/metastatic	Solid tumors	INCAGN02385+INCAGN02390 (Anti-TIM-3)INCMGA00012 (anti-PD-1)	Recruiting
		NCT03311412	1	Advanced/metastatic	Solid tumors or lymphomas	Sym022+Sym021 (anti-PD-1)Sym023 (anti-TIM-3)	Recruiting
	ECO202	NCT03600090	1	Advanced/metastatic	Breast cancer	ECO202+paclitaxel	Recruiting
	89Zr-DFO-REGN3767	NCT04566978	1	Measurable disease by Lugano criteria	DLBCL		Recruiting
	XmAb22,841	NCT03849469	1	Advanced/metastatic	Solid tumors	XmAb22841pembrolizumab	Recruiting
	LBL-007	NCT04640545	1	Advanced/metastatic	Melanoma	LBL-007+toripalimab (anti-PD-1)	Not yet recruiting
	FS118	NCT03440437	1	Advanced/metastatic	Solid or hematologic malignancy		Active, not recruiting
	RO7247669	NCT04140500	1	Advanced/metastatic	Solid tumors		Recruiting
	EMB-02	NCT04618393	1,2	Advanced/metastatic	Solid tumors		Not yet recruiting
**TIGIT**	Tiragolumab (MTIG7192A/RG-6058)	NCT02794571	1	Locally advanced or metastatic	Solid tumors	Tiragolumabatezolizumabchemotherapy	Recruiting
		NCT03563716	2	Locally advanced or metastatic	NSCLC	Atezolizumabtiragolumab	Active, not recruiting
		NCT04294810	3	Locally advanced or metastatic	NSCLC	Atezolizumabtiragolumab	Recruiting
		NCT04256421	3	First line, extensive stage	SCLC	Atezolizumab+carboplatin+etoposidetiragolumab	Recruiting
		NCT03281369	1,2	Advanced/metastatic	Esophageal cancer	Atezolizumab+tiragolumab; atezolizumab+cisplatin/5-FUtiragolumab; cisplatin/5-FU	Recruiting
					GC, GEJ cancer	Atezolizumab+cobimetinib with mFOFLOX6; atezolizumab+cobimetinib or tiragolumab or mFOFLOX or linagliptin or PEGPH20 or BL-8040; pactliaxel+ramucirumab	Recruiting
	Vibostolimab (MK-7684)	NCT02964013	1	Advanced/metastatic	Solid tumors	Vibostolimabpembrolizumab+pemetrexed/carboplatin; carboplatin+cisplatin+etoposide	Recruiting
		NCT04305054	1,2	First line	Melanoma	pembrolizumabvibostolimab or quavonlimab (MK-1308)lenvatinib	Recruiting
		NCT04305041	1,2	Stage III-IV	Melanoma	pembrolizumab+quavonlimab+ vibostolimab or lenvatinib	Recruiting
		NCT04303169	1,2	Stage III	Melanoma	pembrolizumabvibostolimab or V937 (oncolytic virus)	Recruiting
	Etigilimab (OMP-313M32)	NCT03119428	1	Locally advanced or metastatic	Solid tumors	Etigilimabnivolumab	Terminated
	BMS-986207	NCT02913313	1,2	Advanced/metastatic	Solid tumors	BMS-986207nivolumab	Active, not recruiting
		NCT04570839	1,2	Advanced/metastatic	Solid tumors	NivolumabBMS-986207 with COM701 (anti-PVRIG Ab)	Recruiting
	Domvanalimab (AB-154)	NCT03628677	1	Advanced/metastatic	Solid tumors	Dombvanalimab+zimberelimab (AB122, anti-PD-1)	Recruiting
		NCT04262856	2	Locally advanced or metastatic	NSCLC	Zimberelimabdombvanalimabetrumadenant	Recruiting
	ASP-8374	NCT03945253	1	Advanced/metastatic	Solid tumors		Completed
		NCT03260322	1	Advanced/metastatic	Solid tumors	ASP-8374pembrolizumab	Active, not recruiting
	IBI939	NCT04353830	1	Advanced/metastatic	Solid tumors	IBI939sintilimab (anti-PD-1)	Recruiting
		NCT04672369	1	Advanced/metastatic	NSCLC	IBI939sintilimab	Not yet recruiting
		NCT04672356	1	Advanced/metastatic	NSCLC and SCLC	IBI939sintilimab	Not yet recruiting
	BGB-A1217	NCT04047862	1	Advanced/metastatic	Solid tumors	BGB-A1217+tiselizumabchemotherapy	Recruiting
	COM902	NCT04354246	1	Advanced/metastatic	Solid tumors		Recruiting
	M6223	NCT04457778	1	Advanced/metastatic	Solid tumors	M6223bintrafusp alfa (M7824)	Recruiting
**TIM-3**	Sym023	NCT03489343	1	Advanced/metastatic	Solid tumors or lymphomas		Completed
	LY3321367	NCT03099109	1	Advanced/metastatic	Solid tumors	LY3300054 (anti-PD-L1)+LY3321367	Active, not recruiting
		NCT02791334	1	Advanced/metastatic	Solid tumors	LY3300054LY3321367 or abemaciclib or ramucirumab or merestinib	Active, not recruiting
	Cobolimab (TSR-022)	NCT02817633	1	Advanced/metastatic	Solid tumors	Cobolimabnivolumab or TSR-042TSR-033docetaxel	Recruiting
		NCT03307785	1	Advanced/metastatic	Solid tumors	Dostarlimab (TSR-042)TSR-022+chemotherapy^a^; dostarlimab+bevacizumabniraparib or chemotherapy^a^	Active, not recruiting
		NCT03680508	2	BCLC stage B or C	HCC	Cobolimab+dostarlimab	Recruiting
		NCT04139902	2	Neoadjuvant	Melanoma	Cobolimabdostarlimab	Recruiting
	Sabatolimab (MBG453)	NCT02608268	1,2	Advanced/metastatic	Solid tumors	Sabatolimabspartalizumab; decitabine	Active, not recruiting
		NCT03961971	1	Advanced/metastatic	GBM	Sabatolimab+spartalizumab	Recruiting
		NCT04623216	1,2	Received one prior aHSCT	AML	Sabatolimabazacitidine	Not yet recruiting
		NCT03066648	1	Relapse/refractory	AML or high risk MDS	Sabatolimabspartalizumabdecitabine	Recruiting
		NCT03940352	1	Relapse/refractory	AML or high risk MDS	HDM201 (p53-MDM2 inhibitor)+sabatolimab or venetoclax	Recruiting
		NCT03946670	2	IPSS-R intermediate, high, or very high risk	MDS	hypomethylating agentssabatolimab	Active, not recruiting
		NCT04266301	3	IPSS-R intermediate, high, or very high risk for MDS	MDS or CML	Sabatolimab+azacitidine	Recruiting
	INCAGN2390	NCT03652077	1	Advanced/metastatic	Solid tumors		Active, not recruiting
	BMS-986258	NCT03446040	1,2	Advanced/metastatic	Solid tumors	BMS-986258+nivolumab or rHuPH20	Recruiting
	SHR-1702	NCT03871855	1	Advanced/metastatic	Solid tumors	SHR-1702camrelizumab	Unknown
	RO7121661	NCT03708328	1	Advanced/metastatic	Solid tumors		Recruiting
**B7-H3**	Enoblituzumab (MGA271)	NCT01391143	1	Advanced/metastatic	Solid tumors		Completed
		NCT02982941	1	Advanced/metastatic	Pediatric solid tumors		Completed
		NCT02923180	2	Localized intermediate and high-risk	Prostate cancer		Active, not recruiting
		NCT04634825	2	Advanced/metastatic	HNSCC	Enoblituzumab+retifanlimab (anti-PD-1 antibody) or tebotelimab (PD-1 and LAG-3 bispecific DART molecule)	Not yet recruiting
		NCT02381314	1	Advanced/metastatic	Solid tumors	Enoblituzumab+ipilimumab	Completed
		NCT02475213	1	Advanced/metastatic	Solid tumors	Enoblituzumab+pembrolizumab or retifanlimab	Active, not recruiting
		NCT04129320	2,3	Advanced/metastatic	HNSCC	Enoblituzumab+retifanlimab or tebotelimab	Withdrawn
	DS-7300a	NCT04145622	1,2	Advanced/metastatic	Solid tumors		Recruiting
	Orlotamab (MGD009)	NCT02628535	1	Advanced/metastatic	solid tumors		Terminated
		NCT03406949	1	Advanced/metastatic	Solid tumors	Orlotamab+retifanlimab	Active, not recruiting
	131I-Omburtamab	NCT01099644	1	Peritoneal involvement	DSRCT		Active, not recruiting
		NCT00089245	1	Advanced/metastatic	CNS or leptomeningeal cancer		Active, not recruiting
		NCT03275402	2,3	Recurrent	Neuroblastoma, CNS, or leptomeningeal metastases		Recruiting
	124I-Omburtamab	NCT01502917	1	Prior external beam radiotherapy	Gliomas	124I-Omburtamab+external beam radiotherapy (prior to study entry)	Recruiting
	177Lu-DTPA-Omburtamab	NCT04167618	1,2	Recurrent	Medulloblastoma		Not yet recruiting
		NCT04315246	1,2	Advanced/metastatic	Leptomeningeal metastasis from solid tumors		Not yet recruiting
	4SCAR-276	NCT04432649	1	Advanced/metastatic	Solid tumors		Recruiting
	SCRI-CARB7H3	NCT04185038	1	Advanced/metastatic	Pediatric CNS tumors		Recruiting
	B7-H3 CAR-T	NCT04385173	1	Recurrent	GBM	B7-H3 CAR-T+temozolomide	Recruiting
		NCT04077866	1,2	Recurrent	GBM	B7-H3 CAR-Ttemozolomide	Recruiting
	CAR.B7-H3	NCT04670068	1	Advanced/metastatic	Epithelial ovarian cancer	B7-H3 CAR-T+fludarabine+cyclophosphamide	Not yet recruiting
	Second generation 4-1BB B7H3-EGFRt-DHFR	NCT04483778	1	Recurrent	Non-primary CNS solid tumors	Second generation 4-1BB B7H3-EGFRt-DHFRsecond generation 4-1BB CD19-Her2tG	Recruiting
**VISTA**	JNJ-61610588	NCT02671955	1	Advanced/metastatic	Solid tumors		Terminated
	CI-8993	NCT04475523	1	Advanced/metastatic	Solid tumors		Recruiting
	CA-170	NCT02812875	1	Advanced/metastatic	Solid tumors or lymphomas		Completed
**ICOS**	GSK3359609	NCT04428333	1,2	Advanced/metastatic	HNSCC	GSK3359609pembrolizumab+fluouracil-platinum based chemotherapy	Recruiting
		NCT04128696	3	Advanced/metastatic	HNSCC	GSK3359609+pembrolizumab	Recruiting
		NCT03693612	2	Advanced/metastatic	Solid tumors	GSK3359609+pembrolizumab; docetaxel+paclitaxel+cetuximab	Recruiting
	JTX-2011	NCT02904226	1,2	Advanced/metastatic	Solid tumors	JTX-2011+pembrolizumab or nivolumab or ipilimumab	Completed
	MEDI-570	NCT02520791	1	Advanced/metastatic	Lymphoma		Recruiting
	KY1044	NCT03829501	1,2	Advanced/metastatic	Solid tumors	KY1044atezolizumab	Recruiting
**BTLA**	INBRX-106	NCT04198766	1	Locally advanced or metastatic	Solid tumors	INBRX-106+pembrolizumab	Recruiting
	Cudarolimab (IBI101)	NCT03758001	1	Advanced/metastatic	Solid tumors	Cudarolimab+sintilimab (anti-PD-1)	Recruiting
	PF-04518600	NCT02315066	1	Advanced/metastatic	Solid tumors	PF-04518600utomilumab (PF-05082566, anti-TNFRSF9)	Completed
	TAB004 (JS004)	NCT04137900	1	Advanced/metastatic	Solid tumors or lymphomas		Recruiting
		NCT04278859	1	Advanced/metastatic	Solid tumors		Recruiting
		NCT04477772	1	Advanced/metastatic	Lymphoma		Recruiting

**Abbreviations:** AML, acute myeloid leukemia; anti-PD-1, anti-programmed death-1; BCLC, Barcelona Clinic Liver Stage; BTLA, B and T-lymphocyte attenuator; CML, chronic myelogenous leukemia; CNS, central nervous system; CRC, colorectal cancer; DART, dual-affinity re-targeting proteins; DLBCL, diffuse large B cell lymphoma; DSRCT, desmoplastic small round cell tumor; GBM, glioblastoma multiforme; GC, gastric cancer; GEJ, gastroesophageal junction cancer; HCC, hepatocellular carcinoma; HNSCC, head and neck squamous cell carcinoma; ICOS, Inducible T cell costimulator; IDO1i, indoleamine 2,3-dioxygenase-1 inhibitor; IPSS-R, revised international prognostic scoring system; LAG3, lymphocyte-associated gene 3; MDM2, mouse double minute 2 homolog; MDS, myelodysplastic syndrome; MSI-H, microsatellite instability-high; MSS, microsatellite stable; NSCLC, non-small cell lung cancer; PEGPH20, pegylated recombinant human hyaluronidase; RCC, Renal cell carcinoma; rHuPH20, recombinant human hyaluronidase PH20 enzyme; SCLC, small cell lung carcinoma; TIGIT, T cell immunoglobulin and ITIM domain; TIM, T-cell immunoglobulin and mucin domain-3; TNBC, triple negative breast cancer; TNFRSF9, tumor necrosis factor receptor superfamily member 9; VEGF, vascular endothelial growth factor; VISTA, V-domain immunoglobulin suppressor of T cell activation.

**Regimens:** mFOLFOX, oxaliplatin 85mg/m^2^ intravenous (IV), leucovorin 400mg/m^2^ IV, and fluorouracil 2400mg/m^2^ IV over 4648h every 2weeks (Q2W) FOLFIRI, irinotecan 180mg/m^2^ IV, leucovorin 400mg/m^2^ IV, and 5-FU 2400mg/m^2^ IV over 4648h (Q2W).

a Chemotherapy: carboplatin/pemetrexed, carboplatin/nab-paclitaxel, or carboplatin/paclitaxel.

A phase 1 study of eftilagimod alpha (IMP321), an antigen-presenting cell (APC) activator for LAG-3, in combination with pembrolizumab was conducted in 24 patients with metastatic melanoma (NCT02676869) (Atkinson et al., 2020). The primary endpoints were the recommended phase 2 dose (RP2D), safety, and tolerability of the combined agents. The study included cohort A of dose escalation and cohort B of extension, and the patients received subcutaneous pembrolizumab and eftilagimod alpha bi-weekly at doses of 1, 6, or 30mg for up to 6 and 12months for Cohorts A and B, respectively. There was no dose-limiting toxicity (DLT) and the treatment was well tolerated, with the injection site as the most common adverse event (AE). The response to treatment was encouraging, with an overall response rate (ORR) of 33 and 50% for pembrolizumab-refractory cohort A and PD-1 naive cohort B patients, respectively.

Similarly, the combination of eftilagimod alpha and pembrolizumab has been investigated in NSCLC and head and neck squamous cell carcinoma (HNSCC) (NCT03625323) (Peguero et al., 2019). The AIPAC study, a placebo-controlled randomized phase IIb study on eftilagimod alpha (or placebo) with paclitaxel as the first-line treatment in patients with metastatic breast cancer (MBC), is also under investigation (NCT02614833) (Dirix and Triebel, 2019). Preliminary results show that the agent could elicit durable immune responses. Clinical data, including progression-free survival (PFS), ORR, OS, and safety, are all awaiting results.

Relatlimab (BMS-986016), an IgG4 mAb targeting LAG-3, has been investigated in various settings and agents, notably with well-established immune checkpoint inhibitors such as nivolumab and ipilimumab and other novel agents such as indoleamine 2,3-dioxygenase-1 (IDO1) inhibitors, CCR2/5 dual antagonist, and anti-TIGIT. Notably, clinical trials are ongoing for phase II/III in previously untreated metastatic melanoma, in combination with or without nivolumab (NCT03470922), phase II of nivolumab and oxaliplatin-based chemotherapy with or without relatlimab in GC or gastroesophageal junction (GEJ) cancer (NCT03662659), and phase II of relatlimab with nivolumab in mismatch repair deficient (dMMR) cancers resistant to prior PD-1/PD-L1 inhibition (Lipson et al., 2018; Feeney et al., 2019; Bever et al., 2020). Relatlimab is being tested in a wide range of tumor types and settings as front- or second-line treatment, in resectable status, and in stage II/III.

An open label, phase 2 study including 72 patients treated with LAG-525, which is an IgG4 mAb for LAG-3, and spartalizumab (PDR001), an anti-PD-1, for advanced solid tumors and hematologic malignancies showed promising activity, especially in neuroendocrine tumors, small cell lung cancer (SCLC), and diffuse large B-cell lymphoma (DLBCL), with a clinical benefit rate at 24weeks (CBR24) of 0.86, 0.27, and 0.804, respectively, meeting its primary endpoint (NCT03365791) (Uboha et al., 2019). In GEJ cancer, the CBR24 was 0.071, and enrollment was stopped for these subsets of patients. Other tumors such as triple-negative breast cancer (TNBC) (NCT03742349 and NCT03499899) and melanoma (NCT03484923) are ongoing trials in advanced and metastatic settings.

The preliminary results of a phase 1 study on cemiplimab (REGN3767), an mAb for LAG-3, as monotherapy (*n* = 27), and in combination with PD-1 mAb (*n* = 42) was conducted in advanced malignancies (NCT03005782) (Papadopoulos et al., 2019). No DLT was observed with in the monotherapy group, whereas the combination group, during treatment with R3767 3mg/kg every 3weeks (Q3W) + cemiplimab 3mg/kg Q3W, experienced grade 4 elevated creatine phosphokinase levels in addition to grade 3 myasthenia gravis. Overall, both treatments were deemed tolerable; cemiplimab 20mg/kg or 1600mg as a fixed dose of Q3W is ongoing further evaluation as monotherapy and as a combination.

Similarly, BI 754111, an mAb for LAG-3, was also tested with BI 754091 (anti-PD-1) in treatment-refractory solid tumors, in a dose escalation phase 1 study, followed by an expansion phase in microsatellite stable (MSS) CRC and anti-PD1/PD-L1 refractory tumors including NSCLC (NCT03156114) (Johnson et al., 2020). The primary endpoints for dose escalation and dose expansion phase were DLT and the maximum tolerated dose (MTD) and ORR, respectively. Biomarker analysis was performed in MSS CRC refractory to immunotherapy; the patients who responded to these agents with a partial response (PR) or stable disease (SD) had increased treatment-associated IFN- gene signature scores (Bendell et al., 2020). Furthermore, patients with high PD-L1 gene expression in pre-treatment biopsy samples responded better to the treatment. Baseline immunohistochemistry of LAG-3 was not a predictive factor for this subset of patients.

Sym022 (anti-LAG-3) was evaluated as a single agent or in combination with sym021 (anti-PD-1) in phase 1 trials for solid tumors or lymphomas (NCT03311412, NCT03489369, and NCT03489343) (Lakhani et al., 2020). Interim analysis showed that 15 patients who were administered monotherapy and 20 patients under combination treatment, had one unconfirmed PR. Both treatment arms had tolerable safety profiles, with the combination treatment showing one grade 34 immune-related hypophysitis. Further assessments of the pharmacokinetic (PK) and pharmacodynamic (PD) markers and the anti-tumor activity of the monotherapy and combination are awaiting results.

MGD013 is a LAG-3 and PD-1 dual-affinity re-targeting (DART) protein; its safety, tolerability, DLT, MTD, PK/PD, and antitumor activity were analyzed in patients with unresectable and metastatic tumors in a phase 1 study (NCT03219268) (Luke et al., 2020). Fifty patients in the dose-escalation phase and 157 patients in the dose-expansion phase, with 46 and 32% of patients with prior exposure to immunotherapy, respectively, were enrolled. No MTD was reached, and the most common treatment-related adverse events (TRAE), which were fatigue and nausea, were well tolerated. Despite exposure to previous immunotherapy, both cohorts included patients with objective responses. More mature clinical data are awaiting results, and biomarker analysis of LAG-3 and PD-L1 is ongoing.

Other agents that are undergoing clinical trials are: 1) mavezelimab (MK-4280), an IgG4 mAb targeting LAG-3 (NCT03598608, NCT02720068, and NCT03516981); 2) TSR-033, an IgG4 mAb targeting LAG-3 (NCT03250832); 3) INCAGN02385, a Fc engineered IgG1k antibody for LAG-3 (NCT03538028, NCT04370704, and NCT03311412); 4) EOC202, a LAG-3 fusion protein (NCT03600090); 5) 89Zr-DFO-REGN3767, an anti-LAG-3 antibody labeled with 89Zr (NCT04566978); 6) XmAb22841, a bispecific antibody to both LAG-3 and CTLA-4 (NCT03849469); 7) LBL-007, an alphaLAG-3 mAb (NCT04640545), and 8) bispecific antibody to both LAG-3 and PD-L1, which includes agents FS118 (NCT03440437), RO7247669 (NCT04140500), and EMB-02 (NCT04618393) treated as monotherapy or in combination for patients with treatment refractory solid and/or hematologic malignancies.

## TIGIT

TIGIT, previously known as Vstm3, VSIG9, or Washington University cell adhesion molecule (WUCAM), is a protein comprising an extracellular IgV domain and an intracellular domain with a canonical ITIM and an immunoglobulin tyrosine tail (ITT) motif (Table 1) (Yu et al., 2009; Levin et al., 2011). TIGIT expression is tightly restricted to lymphocytes and is mainly observed in NK cells and T cell subsets, including effector and regulatory CD4^+^ T cells, follicular helper CD4^+^ T cells, and effector CD8^+^ T cells (Boles et al., 2009; Yu et al., 2009; Lozano et al., 2012; Stengel et al., 2012; Johnston et al., 2014; Joller et al., 2014). Three ligands bind to TIGIT: 1) poliovirus receptor (PVR), also known as CD155, Necl5, and Tage4; 2) CD112, also called poliovirus receptor ligand2/nectin2 (PVRL2/nectin 2); and 3) PVRL3. PVR has a high affinity for TIGIT, whereas CD112 and PVRL3 bind to a lesser extent (Yu et al., 2009).

TIGIT plays multiple roles in the inhibition of cancer immunity. TIGIT inhibits NK cell-mediated tumor killing, induces immunosuppressive DCs, suppresses CD8 T cell priming and differentiation, and prevents CD8 T cell-mediated killing (Buisson and Triebel, 2005; Li et al., 2014; Fuhrman et al., 2015; Kurtulus et al., 2015; Liu et al., 2015; Kourepini et al., 2016). The interaction of TIGIT with other constituents of the tumor microenvironments (TMEs), such as cancer-associated fibroblasts and angiogenesis, remains to be elucidated (Manieri et al., 2017).

Recently, several studies have highlighted that TIGIT is co-expressed and associated with PD-1 expression (Johnston et al., 2014; Chauvin et al., 2015). Dual blockade of TIGIT and PD-1 resulted in the restoration of T-cell immunity in preclinical settings and provided a rationale for combination with these agents as a feasible anti-cancer therapeutic strategy (Johnston et al., 2014; Kurtulus et al., 2015; Zhang et al., 2018).

### Clinical Trials on TIGIT

Among the 10 anti-TIGIT mAbs undergoing clinical trials, one of the most promising agents is tiragolumab (GO30103) (Table 2). In a randomized, double-blind, phase 2 trial, 135 treatment-nave patients with unresectable and metastatic NSCLC, positive for PD-L1 expression, were treated with tiragolumab (or placebo) in combination with atezolizumab (anti-PD-L1) (NCT03563716) (Rodriguez-Abreu et al., 2020). Primary analysis of CITYSCAPE showed that the result was significant and durable, especially in patients with a PD-L1 tumor proportion score (TPS) 50% in the tiragolumab and atezolizumab groups, with an ORR of 31.3 vs. 16.2% and median PFS of 5.4 and 3.6months in the combination treatment and atezolizumab monotherapy, respectively (hazard ratio 0.57, 95% confidence interval [CI] 0.370.90). The combination was well tolerated and had acceptable safety profiles. The positive and robust results of this trial prompted initiation of phase III in select patients with high PD-L1 expression (SKYSCRAPER-1, NCT04294810). Furthermore, the combination was supplemented with chemotherapy in chemotherapy-naive extensive stage SCLC (SKYSCRAPER-2, NCT04256421). Phase 1 and 2 clinical trials on tiragolumab are also ongoing for esophageal and gastric cancers (NCT03281369) in metastatic settings.

Vibostolimab (MK-7684) is also an anti-TIGIT mAb. The preliminary results of a phase 1 dose-finding study of vibostolimab (200 or 210mg) with pembrolizumab (200mg) on day 1 of each Q3W cycle administered to patients with advanced/metastatic solid tumors without prior anti-PD-1/PD-1, showed acceptable toxicity profiles (NCT02964013) (Niu et al., 2020). The ORR and median PFS were 29% and 5.4months for all patients, and 46% and 8.4months for 13 patients with TPS 1%, respectively. The effects of vibostolimab are also being investigated in melanoma, in combination with other agents (NCT04305054, NCT04305041, and NCT04303169).

Other anti-TIGIT mAbs under investigation include BMS-986207 (NCT02913313 and NCT04570839), domvanalimab (AB-154) (NCT03628677 and NCT04262856), ASP-8374 (NCT03945253 and NCT03260322), IBI939 (NCT04353830, NCT04672369, and NCT04672356), BGB-A1217 (NCT04047862), COM902 (NCT04354246), and M6223 (NCT04457778) as monotherapy or in combination with other agents in the treatment of refractory solid tumors. These agents are being tested in phase 1/2 trials and the results are awaited.

## TIM-3

TIM-3, previously known as hepatitis A virus cellular receptor 2 (HAVCR2), is a member of the TIM gene family, encoding proteins such as TIM-1 and TIM-4 (Table 1) (Monney et al., 2002). It is structured with type-1 cell surface glycoproteins, an extracellular Ig variable region (IgV)-like domain, a mucin-like and transmembrane domain, and an intracellular cytoplasmic tail composed of five tyrosine residues (Monney et al., 2002). Once the two tyrosine residues, Y265 and 272, are phosphorylated by Src kinases or interleukin inducible T cell kinase, the downstream signaling of TIM-3 is activated (van de Weyer et al., 2006; Nagahara et al., 2008).

TIM-3 is expressed in tumor cells and immune cells, such as helper T cells (Th1), IL-17-producing CD4^+^ effector cell lineage (Th17), CD8^+^ T cells, Tregs, TILs, and innate immune cells (Monney et al., 2002; Huang et al., 2010; Jan et al., 2011; Anderson, 2012). Four ligands bind to TIM-3: two soluble ligands, high-mobility group protein B1 (HMGB1) and galectin-9, and two surface ligands, including carcinoembryonic antigen cell adhesion molecule 1 (ceacam-1) and phosphatidyl serine (PtdSer) (Zhu et al., 2005; Nakayama et al., 2009; Chiba et al., 2012; Huang et al., 2015; Kang et al., 2015). Interaction of TIM-3 with its ligands has been shown to induce T cell inhibition. TIM-3 is unique compared to other immune checkpoints in that its upregulation is initiated only by CD4^+^ and CD8^+^ cells that produce IFN- (Sakuishi et al., 2010; Gao et al., 2012).

Similar to PD-L1, TIM-3 is expressed in TILs is associated with disease progression in certain cancers (Ngiow et al., 2011). Meta-analysis of TIM-3 overexpression in solid tumors has shown that higher TIM-3 expression is associated with worse OS and may potentially be a prognostic marker (Zhang et al., 2017). Blocking TIM-3 expression results in T cell proliferation and cytokine production, thereby eliciting immune activation (Gao et al., 2012). In addition, targeting TIM-3 with PD-1 in preclinical settings has shown a synergistic effect by reinvigorating T cell function and increasing anti-tumor immunity (Sakuishi et al., 2010; Koyama et al., 2016). Thus, the dual blockade of PD-1 and TIM-3 is a feasible and promising therapeutic option.

### Clinical Trials on TIM-3

There are seven anti-TIM-3 mAbs and one anti-PD-1 and TIM-3 bispecific Ab (RO7121661) undergoing clinical trials (Table 2). Sym021 (anti-PD-1), sym022 (anti-LAG-3), and sym023 (anti-TIM-3) were evaluated as single agents or combinations in phase 1 trials for solid tumors or lymphomas (NCT03311412, NCT03489369, and NCT03489343) (Lakhani et al., 2020). Sym023 monotherapy (*n* = 24) and in combination with Sym021 (*n* = 17) was administered; however, Sym023 and its combination did not reach their MTD. One patient in the monotherapy group had grade 34 immune-mediated arthritis. Overall, monotherapy and combination therapy were well tolerated, with two PRs observed in the combination group.

LY3321367 is also an anti-TIM-3 mAb; an interim analysis of a phase 1a/1b, dose-escalation and -expansion study showed that intravenous infusion of 31200mg LY3321367 Q2W monotherapy (Arm A, 23 patients) or 701200mg LY3321367 + 200700mg LY3300054 (anti-PD-L1) Q2W combination therapy (Arm B, 18 patients) was well tolerated in the treatment of refractory solid tumors; further, no DLT was observed and most TRAEs observed were grade 2 (NCT03099109) (Harding et al., 2019). Two patients in arm A showed >20% tumor reduction. Overall, there was no effect on the pharmacokinetics, and the antidrug antibody titers were low; thus, Eli Lily dropped the agent from its pipeline.

Other investigational agents targeting TIM-3 include cobolimab (TSR-022), sabatolimab, INCAGN2390, BMS-986258, SHR-1702, and RO7121661, which are currently ongoing clinical trials. Cobolimab is administered in combination with chemotherapy, targeted agents, or immune checkpoints in solid tumors (NCT02817633, NCT03307785, NCT03680508, and NCT04139902). Sabatolimab (MBG453) is administered with other agents in solid tumors (NCT02608268 and NCT03961971) or in acute myeloid lymphoma (AML) (NCT04623216), high-risk myelodysplastic syndrome (MDS) (NCT03066648, NCT03940352, and NCT03946670), and chronic myelogenous leukemia (CML) (NCT04266301). In solid tumors, INCAGN2390 is administered as a monotherapy (NCT03652077), BMS-986258 is administered in combination with nivolumab or rHuPH20 (NCT03446040), SHR-1702 is administered with or without camrelizumab, an anti-PD-1 agent (NCT03871855), and RO7121661, a TIM-3 bispecific Ab, is administered as a monotherapy (NCT03708328).

## B7-H3

B7-H3, also called CD276, is a member of the B7 family. It was initially recognized as a co-stimulatory molecule that activates T cells and IFN- production (Table 1) (Chapoval et al., 2001). B7-H3 is found in activated immune cells such as antigen-presenting cells (APCs), NK cells, T cells, and monocytes (Janakiram et al., 2017). In addition, B7-H3 is expressed in several tumors. Notably, high levels of B7-H3 expression in NSCLC, RCC, CRC, and prostate cancer are correlated with disease progression (Li et al., 2014; Jin et al., 2015; Benzon et al., 2017; Mao et al., 2017). In NSCLC, B7-H3 with Tregs was associated with poor prognosis, and co-expression of B7-H3 and CD14 was found to play a role in angiogenesis and tumor progression in RCC (Li et al., 2014; Jin et al., 2015). Patients with CRC, harboring B7-H3 and CD133 expression, have shorter survival (Castellanos et al., 2017). Similarly, high levels of B7-H3 are associated with higher Gleason grade, advanced stage, and poor outcomes in prostate cancer (Benzon et al., 2017).

Recently, the co-inhibitory function of B7-H3 in CD4^+^ and CD8^+^ T cells was discovered (Suh et al., 2003; Prasad et al., 2004). Studies are ongoing to identify the receptor for B7-H3, and the contradictory roles of B7-H3 in immune activity are yet to be fully elucidated (Yang et al., 2020). In addition to the immunological aspects of B7-H3, other signaling pathways, including PI3K/AKT/mTOR, JAK2/STAT3, and TLR4/NF-B signaling, can activate B7-H3 expression (Kang et al., 2015; Zhang et al., 2015; Xie et al., 2016; Fan et al., 2017; Zhang et al., 2017). Other studies have highlighted that B7-H3 is associated with resistance to chemotherapy and targeted agents (Liu et al., 2011; Jiang et al., 2016; Flem-Karlsen et al., 2017; Flem-Karlsen et al., 2019).

### Clinical Trials on B7-H3

Eleven agents targeting B7-H3 are currently under investigation in clinical trials (Table 2). Generally, patients harboring B7-H3 are enrolled in clinical trials. Enoblituzumab (MGA271), an anti-B7-H3 mAb with antibody-dependent cellular toxicity (ADCC) function, has been investigated in multiple solid tumors, including pediatric tumors. Interim analysis of enoblituzumab in refractory solid tumors revealed that it was well tolerated up to 15mg/kg, with no DLT and MTD (Powderly et al., 2015). Although TRAEs, such as fatigue (30%) and infusion-related reactions (26%), occurred in 71% of the patients, most of these AEs were tolerated with adequate supportive care (NCT01391143). Enoblituzumab is currently being used as a monotherapy or in combination with anti-PD-1 antibody (retifanlimab or pembrolizumab), tebotelimab, a PD-1 and LAG-3 bispecific DART, or ipilimumab, as shown in Table 3.

DS-7300a is a B7-H3-targeting antibody drug conjugate (ADC) with DXd, a payload that is an exatecan derivative, which inhibits topoisomerase I (Bendell et al., 2020). The phase 1/II study is ongoing with patients enrolled in the dose-escalation part (NCT04145622). Orlotamab (MGD009) is a B7-H3 and CD3 DART protein, and its monotherapy (NCT02628535) and combination with retifanlimab (NCT03406949) are under investigation in heavily treated solid tumors. Orlotamab with radioactive labeling such as 131I-Omburtamab (NCT01099644, NCT00089245, and NCT03275402), 124I-Omburtamab (NCT01502917), and 177Lu-DTPA-Omburtamab (NCT04167618 and NCT04315246) are also ongoing trials. In patients with desmoplastic small round cell tumor (DSRCT), treatment with 131I-Omburtamab via intraperitoneal administration followed by external beam intensity-modulated whole-abdominopelvic radiotherapy (WAP-IMRT) to 3,000cGy was tolerable with a satisfactory safety profile, and appeared to demonstrate micro-metastatic activity in a phase 1 trial (Modak et al., 2018). The biodistribution, organ, and whole-body exposure were measured with 124I-8H9-directed radioimmuno-PET, and the RP2D for 131I-Omburtamab was set at 80mCi/m^2^.

Other investigational agents include chimeric antigen receptor (CAR) T cell therapy targeting B7-H3: 4SCAR-276 in solid tumors (NCT04432649), SCRI-CARB7H3 in pediatric CNS tumors (NCT04185038), B7-H3 chimeric antigen receptor T cells (CAR-T) treated alone (NCT04385173) or with temozolamide (NCT04077866) in glioblastoma, CAR.B7-H3 with other agents in epithelial ovarian cancer (NCT04670068), and second-generation 4-1BB B7H3-EGFRt-DHFR in non-primary CNS solid tumors (NCT04483778).

## VISTA

VISTA has several names such as differentiation of embryonic stem cells 1 (Dies1), DD1 , Gi24, and B7H5 (Table 1). (Ceeraz et al., 2013). Notably, it is also named PD-1 homologue (PD-1H), as its extracellular domain shows structural similarity to PD-1; however, it is different, as it lacks the classical ITIM or ITSM motif in the cytoplasmic domain (Flies et al., 2011). Furthermore, VISTA differs from PD-1, which functions in the effector stage, as VISTA is expressed on resting T cells, indicating its regulatory role in earlier stages (Kondo et al., 2016). Compared to that in peripheral lymph nodes, VISTA is more abundant in myeloid-derived suppressor cells (MDSCs) in the tumor microenvironment (TME) (Le Mercier et al., 2014).

High levels of VISTA are expressed by mature APCs with CD11b, whereas relatively low expression is found on Tregs, CD8^+^, CD4^+^, and TILs (Lines et al., 2014). Although the counter structures for VISTA have not been comprehensively elucidated, recent *in vitro* findings on V-Set and immunoglobulin domain containing 3 (VSIG-3) have shown that VISTA also acts as a co-inhibitory ligand on tumor cells (Wang et al., 2019). VISTA promotes Treg maturation and prevents T cell activation independent of PD-1 expression (Yoon et al., 2015; Torphy et al., 2017; Popovic et al., 2018). The non-overlapping mechanisms of VISTA and PD-L1 make their combination an ideal treatment strategy to overcome immune suppression. In mouse models, dual blockade of VISTA and PD-1, using monoclonal antibodies specific for these immune checkpoints, led to synergistic activity against T-cells with anti-tumor responses (Liu et al., 2015).

A wide array of tumors has been studied to determine the prognostic and predictive roles of VISTA. High-grade serous ovarian cancer patients with tumor cells expressing VISTA showed longer PFS and OS (Zong et al., 2020). Furthermore, VISTA expression on TILs in pT1/2 esophageal adenocarcinoma was associated with improved OS compared to the TILs negative for VISTA (Loeser et al., 2019). Similarly, VISTA^+^ and CD8^+^ TIL subtypes are associated with better OS in HCC (Zhang et al., 2018). Contrary to these findings, VISTA^+^ and CD8^+^ TIL subtypes were associated with worse prognosis in oral squamous cell carcinoma and cutaneous melanoma with VISTA expression, whereas VISTA had no correlation with survival outcome in GC expressing VISTA(Bger et al., 2017; Wu et al., 2017; Kuklinski et al., 2018).

### Clinical Trials on VISTA

Ongoing clinical trials on VISTA include two anti-VISTA mAbs and one small-molecule antagonist of VISTA (Table 2). JNJ-61610588 (NCT02671955) and CI-8993 (NCT04475523) are anti-VISTA mAbs, currently under investigation in phase 1 trials for the treatment of refractory solid tumors. CA-170 is a small molecule that targets both VISTA and PD-L1 (Musielak et al., 2019). A phase 1 study in patients with advanced solid tumors or lymphomas showed no DLT during dose escalation in 19 patients treated across six dose levels (50800mg) (NCT02812875) (Powderly et al., 2017). Exploratory analysis showed an increased proportion of both circulating CD8^+^ and CD4^+^ cells after oral dosing with CA-170. Further data on dose escalation, the recommended phase 2 dose, and anti-tumor responses are awaiting results.

## ICOS

ICOS, also known as cluster of differentiation 278 (CD278) in T cells, is a member of the CD28 coreceptor family, which includes costimulatory CD28 and coinhibitory receptor CTLA-4 (Table 1) (Hutloff et al., 1999). The ICOS ligand (ICOSL) is expressed in APCs such as macrophages, DCs, and B cells (Yoshinaga et al., 1999). In contrast to the expression of CD28 in both naive and memory T cells, the majority of ICOS is expressed only after the activation of memory T cells, with only small fractions expressed in resting memory T cells. Further, unlike CD28 and CTLA-4 ligands, which are expressed primarily on lymphoid tissues, ICOSL is expressed in non-lymphoid cells, such as endothelial cells, epithelial cells, mesenchymal cells, and fibroblasts, via the activation of tumor necrosis factor- (Swallow et al., 1999; Khayyamian et al., 2002; Martin-Orozco et al., 2010). Activation of the ICOS pathway induces the production of cytokines, such as IL-4, IL-10, and IL-21, by CD4^+^ Th cells, CD4^+^ forkhead box P3 (FoxP3^+^) Tregs, and CD8^+^ cytotoxic T lymphocytes (CTL) (Hutloff et al., 1999; Gigoux et al., 2009; Solinas et al., 2020). ICOS interacts with its ligand (ICOSL) to increase anti-tumor effects via the regulation of memory and effector T cell development and humoral immune responses (Marinelli et al., 2018). The rationale for targeting the ICOS/ICOSL axis with agonists and antagonists is its capacity to trigger both anti-tumor T cell responses by Th1 and other effector T cells, as well as its protumor responses via Tregs (Solinas et al., 2020).

In preclinical studies, ICOS expression on FoxP3^+^ Tregs and other Th subsets has been identified in multiple arrays of solid tumors, including melanoma, gastric, colorectal, and breast cancers (Strauss et al., 2008; Zhang et al., 2016; Gu-Trantien et al., 2017; Nagase et al., 2017). ICOS^+^ Treg TILs have been found to be associated with worse survival in GC, whereas high levels of ICOS in Th1 TILs in colorectal cancer indicated better survival outcomes (Zhang et al., 2016; Nagase et al., 2017). Dual blockade of ICOS with anti-CTLA-4 has been effective in eliciting anti-tumor responses in ICOS knockout mice that were unresponsive to anti-CTLA-4 monotherapy (Fu et al., 2011; Fan et al., 2014). More importantly, the utilization of ICOS-targeted agents is gaining attention in hematological malignancies owing to the enhancement of co-stimulatory receptor 4-1BB in CD4^+^ CAR T cells by ICOS (Guedan et al., 2018).

### Clinical Trials on ICOS

Currently, both anti-ICOS agonists and anti-ICOS antagonists are under clinical investigation (Table 2). The phase 1 trial of GSK3359609 (INDUCE-1), a humanized anti-ICOS agonist monoclonal antibody, comprised two treatment groups: part 1 patients were treated with a monotherapy of GSK3359609, and part 2 patients were administered a combination with pembrolizumab or other immunotherapy in the treatment of advanced solid tumors. The study is ongoing, with no dose-limiting toxicities from the first three dose-limiting cohorts (Angevin et al., 2017). In head and neck cancer, the efficacy of GSK3359609 and pembrolizumab with or without platinum-based chemotherapy is currently under investigation (NCT04428333 and NCT04128696).

Another investigational anti-ICOS agonist monoclonal antibody is JTX-2011, used in combination with either anti-PD1 (pembrolizumab or nivolumab) or anti-CTLA-4 (ipilimumab) in advanced solid tumors (NCT02904226) (Yap et al., 2018). In phase 1/II of the trial, anti-tumor activity was observed with JTX-2011 monotherapy and in combination with nivolumab, in heavily treated GC and TNBC with manageable toxicity profiles. Exploratory analysis showed that the peripheral blood CD4 ICOS^high^ T cell subsets may be a potential biomarker for the response.

Further, agonistic antibodies such as MEDI-570 alone and KY1044 with atezolizumab are under investigation in phases 1 and phase 1/II, respectively (NCT02520791 and NCT03829501).

## BTLA

BTLA (CD272) is also a member of the CD28 coreceptor family (Table 1) (Ceeraz et al., 2013). It is a co-inhibitory molecule with a structure and function similar to those of PD-1 and CTLA-4 (Paulos and June, 2010). When expressed on mature lymphocytes, such as B cells and T cells, macrophages, and DCs, BTLA binds to herpes virus entry mediator (HVEM), a member of the tumor necrosis factor receptor superfamily (TNFRSF), as well as to LIGHT and lymphotoxin-, two members of the tumor necrosis factor (TNF) superfamily (Han et al., 2004; Sedy et al., 2005; Steinberg et al., 2011). Binding of BTLA to HVEM via CD160 transmits inhibitory signals to T cells, which are necessary for proliferation and cytokine production, whereas binding to LIGHT induces co-stimulatory signals (Sedy et al., 2005; Murphy et al., 2006; Cai et al., 2008). Thus, the complexity of the BTLA receptor and ligand activity poses a challenge for BTLA blockade treatment.

Recently, the possibility of BTLA as a potential therapeutic target in cancer immunotherapy has been established *in vivo,* wherein human melanoma tumor antigen-specific effector CD8^+^ T cells expressing high levels of BTLA were downregulated with a vaccine formulated using CpG oligodeoxynucleotides, a toll-like receptor 9 (TLR9) agonist that triggers innate immunity, thereby proving that inhibition of BTLA may partially reverse the function of human CD8^+^ cancer-specific T cells (Derr et al., 2010; Paulos and June, 2010).

### Clinical Trials on BTLA

There are four agents targeting BTLA (Table 2): 1) INBRX-106, a hexavalent OX40 agonist Ab (NCT04198766), 2) PF-04518600 (NCT02315066), an OX40 agonist; 3) cudarolimab (IBI101) (NCT03758001), an anti-OX40 mAb, and 4) TAB004 (JS004) (NCT04278859), an anti-BTLA mAb. These agents target the OX40 receptor, also known as CD134 and tumor necrosis factor receptor superfamily member 4 (TNFRSF4), thereby preventing its interaction with BTLA (Croft et al., 2009). These phase 1 clinical trials are ongoing as monotherapy for patients with advanced/metastatic solid tumors and are awaiting results. TAB004 is also under investigation for the treatment of refractory lymphomas (NCT04137900 and NCT04477772).

## Conclusion

Cancer immunotherapy is one of the major pillars in the field of medical oncology, especially for the treatment of unresectable, metastatic, and recurrent cancers. The success of ICIs, such as anti-CTLA-4 and anti-PD-1/PD-L1, in combination with chemotherapy, immunotherapy, and targeted agents, has changed the paradigm of cancer treatment. Nonetheless, the limited efficacy and IRAEs of ICIs have paved way for the discovery of novel checkpoints. Among the immune checkpoint inhibitors, anti-LAG-3 and anti-TIGIT are promising targets, and their efficacy in combination with anti-PD-1/PD-L1 may help overcome the limitations seen in prior treatments. More robust data are yet to follow on agents targeting TIM-3, B7-H3, VISTA, ICOS, and BTLA.

## Abbreviations

adhesion molecule 1; BTLA, B and T-lymphocyte attenuator; HVEM, herpes-virus entry mediator; ICOS, Inducible T cell costimulator; ICOSL, Inducible T cell costimulatory ligand; LAG-3, lymphocyte-associated gene 3; mAb, monoclonal antibody; PtdSer, phosphatidyl serine; TIGIT, T cell immunoglobulin and ITIM domain; TIM-3, T-cell immunoglobulin and mucin domain-3; VISTA, V-domain immunoglobulin suppressor of T cell activation; VSIG-3, V-Set and Immunoglobulin domain containing 3.
